# Alcelaphine Herpesvirus-1 (Malignant Catarrhal Fever Virus) in Wildebeest Placenta: Genetic Variation of ORF50 and A9.5 Alleles

**DOI:** 10.1371/journal.pone.0124121

**Published:** 2015-05-13

**Authors:** Felix Lankester, Ahmed Lugelo, Nicholas Mnyambwa, Ahab Ndabigaye, Julius Keyyu, Rudovick Kazwala, Dawn M. Grant, Valerie Relf, David M. Haig, Sarah Cleaveland, George C. Russell

**Affiliations:** 1 Boyd Orr Centre for Population and Ecosystem Health, Institute of Biodiversity, Animal Health & Comparative Medicine, University of Glasgow, Glasgow, G12 8QQ, United Kingdom; 2 Paul G. Allen School for Global Animal Health, Washington State University, Pullman, WA, 99164, United States of America; 3 School of Life Sciences and Bioengineering, Nelson Mandela African Institution of Science and Technology, Arusha, Tanzania; 4 Faculty of Veterinary Medicine, Sokoine University of Agriculture, Morogoro, Tanzania; 5 Department of Science and Laboratory Technology, Dar es Salaam Institute of Technology, Dar es Salaam, Tanzania; 6 Tanzania Wildlife Research Institute, Arusha, Tanzania; 7 Moredun Research Institute, Pentlands Science Park, Penicuik, Edinburgh, United Kingdom; 8 School of Veterinary Medicine and Science, University of Nottingham, Sutton Bonington, Leicestershire, United Kingdom; Cornell University, UNITED STATES

## Abstract

Alcelaphine herpesvirus–1 (AlHV-1), a causative agent of malignant catarrhal fever in cattle, was detected in wildebeest (*Connochaetes taurinus*) placenta tissue for the first time. Although viral load was low, the finding of viral DNA in over 50% of 94 samples tested lends support to the possibility that placental tissue could play a role in disease transmission and that wildebeest calves are infected *in utero*. Two viral loci were sequenced to examine variation among virus samples obtained from wildebeest and cattle: the ORF50 gene, encoding the lytic cycle transactivator protein, and the A9.5 gene, encoding a novel polymorphic viral glycoprotein. ORF50 was well conserved with six newly discovered alleles differing at only one or two base positions. In contrast, while only three new A9.5 alleles were discovered, these differed by up to 13% at the nucleotide level and up to 20% at the amino acid level. Structural homology searching performed with the additional A9.5 sequences determined in this study adds power to recent analysis identifying the four-helix bundle cytokine interleukin-4 (IL4) as the major homologue. The majority of MCF virus samples obtained from Tanzanian cattle and wildebeest encoded A9.5 polypeptides identical to the previously characterized A9.5 allele present in the laboratory maintained AlHV-1 C500 strain. This supports the view that AlHV-1 C500 is suitable for the development of a vaccine for wildebeest-associated MCF.

## Introduction

Malignant catarrhal fever (MCF) is a fatal systemic disease of a range of species—mainly ungulate—that is caused by members of a group of related herpesviruses, belonging to the genus *Macavirus* in the sub-family *gammaherpesvirus*. Although the best studied members of this genus are ovine herpesvirus-2 (OvHV-2) [1],[2] and alcelaphine herpesvirus-1 (AlHV-1) [1],[3], MCF has also been associated with infection by caprine herpesvirus-2 (CpHV-2) [4],[5], the MCF virus of white-tailed deer (*Odocoileus virginianus)* (MCFV-WTD) [6],[7] and alcelaphine herpesvirus-2 (AlHV-2) [8],[9],[10]. These viruses share contrasting abilities to propagate infectious virus without inducing clinical signs within specific reservoir host species (sheep for OvHV-2; wildebeest (*Connochaetes taurinus*) for AlHV-1; Topi *(Damaliscus lunatus)* for AlHV-2; and goat for CpHV-2) and also to induce fatal MCF, without the production of infectious virus, in selected MCF-susceptible species including bison *(Bison bonasus)*, cattle, deer and others [11]-[12].

Reservoir species generally shed MCF virus in ocular and nasal secretions on an irregular basis, in keeping with the latent nature of herpesvirus infections [13],[14]. The ability to induce MCF in susceptible species (and by inference to shed infectious virus) appears to be limited to periods of detectable viraemia in the reservoir species. For AlHV-1, it has been suggested that viraemia in wildebeest calves is at its highest in the first three months of life, and thereafter shedding ceases [15]. For OvHV-2, virus appears to be shed intermittently in nasal secretions of latently infected sheep, with adolescents representing the highest risk group for transmission [16].

Experimentally, MCF can be induced and infectivity maintained by inoculation of infected cells in to susceptible hosts [17,18] or by intranasal inoculation with cell-free virus [19,20]. Natural transmission of MCF viruses, however, is generally considered to be *via* inhalation or ingestion of infectious cell-free virus shed in the mucous secretions of reservoir hosts. The induction of MCF in cattle following inoculation with tissue suspensions from wildebeest foetal liver or spleen, however, suggested that intrauterine transmission of AlHV-1 could also occur. This was supported by the detection of virus in the spleen of one wildebeest foetus and in the blood of three wildebeest calves less than one week old [3].

Tanzanian pastoralists view the calving wildebeest as the main source of MCF, with MCF transmission from placenta, foetal membranes, foetal fluid or calf hair (moulted at 3–4 months) considered a high risk [21]. These Maasai perceptions of placental involvement in MCF transmission are firmly held but are not supported by the available evidence despite demonstrations of apparent intra-uterine infection of wildebeest calves [3],[15],[22],[23]. The availability of diagnostic PCR now makes it possible to analyse placental tissue samples for the presence of AlHV-1.

The novel spliced gene A9.5 is predicted to encode a secreted glycoprotein that has no homologue outside the MCF viruses [24]. A9.5 does have a detectable homologue in OvHV-2 (Ov9.5) that has been shown to be both polymorphic and highly variable with nine alleles showing as little as 50% predicted amino acid sequence identity [25]. So far, only two alleles of A9.5 have been identified: one (A9.5*0101] [24] in the C500 strain of AlHV-1, that has been passaged *in vivo* in the UK for almost 50 years [26], and the other (A9.5*0201) [27] in a diagnostic sample taken from an Ankole cow with MCF. The A9.5 alleles share about 80% amino acid sequence identity and retain overall similarity to the Ov9.5 alleles. It is expected that analysis of this gene in a range of samples from wildebeest and cattle with MCF will allow improved epidemiological analysis of MCF. Of particular interest for this study is the potential for comparative analysis of AlHV-1 from two wildebeest populations in East Africa that have been geographically isolated from each other [28]. Of added interest will be the comparison of these strains with the C500 virus which has been the strain of choice for recent vaccine development [20,29].

MCF reservoir species frequently have neutralising antibodies that react with AlHV-1 and appear to share a specific epitope in the major virus glycoprotein complex [30]. Thus, serological analysis of wildebeest blood samples might be used to detect past infection even when latent virus levels in the blood are below the limit of detection by PCR.

In this paper we describe the first analysis by PCR of AlHV-1 DNA from wildebeest placental samples and DNA sequence-based analysis of virus diversity among AlHV-1 samples from both wildebeest and cattle.

## Materials and Methods

Biological samples for analysis of AlHV-1 infection were collected from four sources and are detailed in Table 1:

**Table 1:** Details of each sample set and summarized results of diagnostic assays.

Animal	Population / Location	Sample	Sample Numbers	Real time PCR positive	Nested PCR positive	AlHV-1 specific antibody positive	A9.5 sequences obtained
Wildebeest	Ngorongoro Crater	Placenta	94	1	50	NA	23
Wildebeest dam	Ngorongoro Crater	Buffy coat	3	0	1	3	0
Wildebeest calf (32 day old)	Ngorongoro Crater	Buffy coat	5	0	5	4	3
Wildebeest calf (100 day old)	Tarangire	Buffy coat	5	0	1	5	1
Cattle (adult)	Tarangire	Buffy coat	200	0	59	154*	6

* This number includes 54 out of 100 *unvaccinated* animals with at least one sample of nasal secretion or blood plasma with MCF-specific ELISA titre >10. Of these animals, 20 were found to be AlHV-1positive by nested PCR.

### i. Wildebeest calf and dam

Uncoagulated blood samples were collected from two wildebeest populations resident in Tanzania: i. The Ngorongoro Crater population, which despite residing in the Crater for long periods of time are known to occasionally disperse and mingle with the population resident in the wider Serengeti ecosystem; ii. The Tarangire population, which live predominantly in the vicinity of Tarangire National Park. The two populations are separate, both in the geographical and evolutionary sense [28]. All sampled wildebeest were immobilized using etorphine hydrochloride (Captivon, Wildlife Pharmaceuticals, Karino, South Africa) and all blood samples were collected by jugular venipuncture directly in to Vacutainer collection tubes. Samples from three dams and five approximately 32-day-old calves were collected from the Ngorongoro Crater population and from five approximately 100-day-old calves from the Tarangire population. The ages of the calves could be accurately judged as the wildebeest calving season, for which the authors recorded the start date, is highly synchronised and only lasts a few days. Buffy coat cells prepared from the blood samples were stored on FTA cards prior to DNA extraction, while blood plasma was stored at minus 20°C.

### ii. Wildebeest placenta

Samples of freshly passed wildebeest placenta from the Ngorongoro Crater population, consisted of approximately 1 cm^3^ of full thickness tissue. To collect these samples field teams followed calving wildebeest waiting for the placenta to be expelled. As soon as the placenta fell away from the dam, approximately 45 minutes after the calf was born, the team would drive up to the placenta and collect the tissue sample. None of the dams paid any attention to their placenta, and there was no licking or sniffing observed. Consequently we consider all samples to have been uncontaminated with the dam’s ocular or nasal secretion. All samples once collected were frozen (at minus 20°C) prior to DNA extraction.

### iii. MCF vaccine trial cattle

Blood samples, collected by jugular venipuncture directly in to Vacutainer collection tubes as part of a vaccine trial involving Tanzanian zebu shorthorn cattle living near Tarangire National Park, were selected for analysis if they came from individuals that had become naturally infected with MCF virus transmitted by wildebeest calves. Buffy coat cells, prepared from the blood samples, were stored on FTA cards (Whatman FTA Classic, Thermo Fisher, UK) or frozen (at minus 20°C) prior to DNA extraction.

### iv. C500

The C500 strain of AlHV-1, for which complete genome sequence is available [31], was originally obtained from an AlHV-1-infected ox in Kenya and passaged in New Zealand White rabbits [26] in the UK. Purified AlHV-1 C500 DNA was used as a control in PCR and sequencing analyses.

### AlHV-1 specific antibody reactivity

Duplicate samples of wildebeest blood plasma were tested twice using an AlHV-1 specific direct ELISA, essentially as described previously [29], except that known positive and negative wildebeest serum samples from the UK were used as controls and to generate standard curves for determination of AlHV-1-specific antibody titre.

### Detection of viral DNA

The presence of AlHV-1 was determined by PCR analysis of DNA extracted from buffy coat cells and placenta tissue samples. DNA was purified from the frozen cattle buffy coat samples using the ZR Viral DNA Kit (Zymo Research Corporation, USA); from the FTA card stored wildebeest buffy coat samples using QiaAmp Mini Kit (Qiagen, Crawley, UK); and from the frozen placental samples using the Qiagen DNeasy Blood and Tissue Kit. Thereafter viral DNA was detected and analysed by AlHV-1-specific duplex real-time PCR [32],[29] (targeting the AlHV-1 ORF3 gene [32] and the genomic β-actin gene [29]) and nested diagnostic PCR [33]. Each set of assays was controlled by the inclusion of known AlHV-1 positive and negative bovine genomic DNA samples and potential cross-contamination between reactions was controlled by the inclusion of template-free reactions. To study viral variation in positive samples, segments of the ORF50 gene, encoding the lytic cycle trans-activator protein, and of the spliced A9.5 gene [24] were sequenced and analysed as described previously for OvHV-2 [25]. ORF50 encodes a transcription factor expected to be highly conserved, whilst A9.5 encodes a predicted glycoprotein of unknown function that has been demonstrated to be polymorphic in both AlHV-1 and OvHV-2 [25],[24].

### PCR amplification of A9.5 and ORF50 genes

Primers for nested PCR of the A9.5 gene were designed to target conserved areas flanking the predicted coding region, based on the available sequence of AlHV-1 [31],[24]. Primers for amplification of ORF50 (AHVorf50_F, GCC AGG CAG AGG TAT GTG TT and AHVorf50_R, GGC CGT TGT GGG TAC TGT AT) were chosen within exon 2 of the ORF50 gene, to amplify a fragment of 543 base pairs for analysis of sequence variation. Primer pairs were designed using Primer3 (www.bioinformatics.nl/cgi-bin/primer3plus/primer3plus.cgi) [34]. The amplification of A9.5 used a nested PCR strategy described in an earlier publication because a non-nested approach amplified the gene poorly. This lack of efficiency probably reflects the constraints on the primer design aimed to amplify the entire A9.5 coding region. In contrast, the primer design for ORF50 required only amplification of a fragment of the gene. These primers amplified the ORF50 segment efficiently without the need for a nested approach.

Amplification of ORF50 and A9.5 gene segments was attempted from genomic DNA samples in which AlHV-1 DNA had been detected by diagnostic nested PCR [33]. For A9.5, a nested PCR approach was used with initial amplification performed in 25 μl reactions using 1 unit KOD Hot Start DNA polymerase (Merck, Feltham, UK), 50–100 ng of genomic DNA, and 5 pmol each of primers A9.5geneF and A9.5ex6R [24]. Amplification reactions consisted of a denaturation / activation step at 94°C for 120 s; 30 cycles of 94°C for 30 s, 55°C for 30 s and 70°C for 60 s; and a final extension step at 70°C for 5 minutes. Aliquots of 2 μl from this initial PCR were then used as templates in nested PCR amplifications using the same enzyme, buffer and PCR conditions but with 10 pmol per reaction of the nested primers A9.5cdsF and A9.5cdsR [24]. The AlHV-1 ORF50 segment was amplified in a single reaction using the same conditions as described above for A9.5 except that 10 pmol per reaction of the AHVorf50_F and AHVorf50_R primers were used and reactions were amplified using 40 cycles of 94°C for 30 s, 59°C for 30 s and 68°C for 60 s.

The PCR products were analysed by agarose gel electrophoresis, stained with SYBR Safe DNA Gel Stain (Life Technologies, Paisley, UK) and visualized by UV transillumination before purification (QIAamp PCR purification system). PCR product concentrations were estimated after purification using a Nanodrop spectrophotometer (Labtech, Uckfield, UK). Approximately 300 ng of each PCR product was submitted for bidirectional nucleotide sequencing by Eurofins MWG Operon (Ebersberg, Germany), using the internal PCR primers as the sequencing primers. Electropherograms from each pair of sequencing reactions were assembled to produce sample consensus sequences for each gene segment amplified.

To confirm that direct sequencing of PCR products produced an accurate representation of the target sequence *in vivo*, PCR products of A9.5 from 4 samples were cloned into pGEM-T-Easy (Promega, Southampton, UK) and at least three clones representing each PCR product were sequenced.

### Bioinformatics

Unless otherwise indicated, all DNA sequence analysis was carried out using DNASTAR Lasergene software (V8.0 and above; www.DNASTAR.com). DNA sequence information from each amplicon was assembled using the SEQMAN program and consensus sequences representing the region flanked by the PCR primers were derived. Any DNA sample that did not give good quality sequence traces on both strands was discarded.

The consensus sequences for each sample and locus were aligned using the MUSCLE algorithm [35] or MAFFT [36]. For A9.5, the positions of introns and exons were defined according to the annotation of A9.5*01 [24] and conservation of splice donor and acceptor sequences was confirmed by visual inspection, while the conservation of a continuous A9.5 open reading frame was confirmed by generation of predicted A9.5 cDNA sequences and their translation. Phylogenetic and evolutionary analysis of all sequences was done by maximum likelihood methods using the program MEGA (version 5 or above; megasoftware.net) [37].

### Nucleotide sequence accession numbers

Nucleotide sequences of the gene fragments amplified in this work have been submitted to the European Nucleotide Archive (ENA; www.ebi.ac.uk/ena) and have been assigned accession numbers as follows: AlHV-1 orf50*0101, LN823968; orf50*0102, LN823969; orf50*0103, LN823970; orf50*0201, LN823971; orf50*0301, LN823972; orf50*0401, LN823973; A9.5*0202, LN823974; A9.5*0203, LN823975; and A9.5*0301, LN823976.

### Approval

The animal ethics committees of the Tanzanian Wildlife Research Institute (TAWIRI) and the Commission for Science and Technology (COSTECH, Tanzania) approved all aspects of this study, including all sampling procedures and the animal research that was conducted according to international guidelines (permit nos.2011-213-ER-2005-141 and 2012-318-ER-2005-141). The Ngorongoro Conservation Area Authority and the Tanzanian National Parks Association approved immobilization (using etorphine hydrochloride loaded pressure darts) and sampling of wildebeest in the Ngorongoro Crater and Tarangire populations respectively.

### Ethical clearance

Permission to immobilise wildebeest, to collect placenta and to carry out the MCF vaccine trial was provided by the Tanzanian Wildlife Research Institute and the Tanzanian Commission for Science and Technology (permit numbers 2010-259-NA-2005-141 and 2011-213-ER-2005-141). Permission to import inactivated blood plasma, serum and DNA on FTA cards into Scotland was provided by the Scottish Executive Rural Directorate (import licenses POAO(S)/2011/54, and POAO(S)/2012/37).

## Results

### Detection of AlHV-1 virus DNA in wildebeest samples

PCR analysis of DNA samples was done by real-time and nested diagnostic PCR assays. The duplex real-time PCR assay detected AlHV-1 DNA in none of the wildebeest buffy coat samples and in only one of the placental samples. All samples showed amplification of the β-actin internal control [29] and therefore were suitable for PCR. In contrast, the more sensitive nested PCR method [33] detected viral DNA in buffy coat samples from one of the three dams, in six of the ten calves and in 50 of the placental samples (summarised in Table 1). The six positive wildebeest calf buffy coat samples consisted of all five of the 32-day old calves from the Ngorongoro Crater population and one of the five 100-day old calves from the Tarangire population.

### Antibody reactivity of wildebeest blood samples

AlHV-1 specific antibody titre in blood plasma was measured in all collected wildebeest blood samples. Results are summarised in Table 1. All but one of the animals tested were positive for AlHV-1 specific antibodies, with titre values >100. This included two wildebeest dams and four 100-day-old calves that tested negative for viral DNA by nested PCR. Interestingly, the single animal that did not have a positive anti-viral antibody titre was a 32-day-old calf that tested positive for viral DNA by nested PCR.

### Sequencing of AlHV-1 loci from wildebeest and cattle

In order to look at viral sequence variation within the AlHV-1 positive samples identified, PCR fragments representing the variable A9.5 locus were analysed. To determine whether variation at A9.5 represented a generally high level of sequence variation between AlHV-1 strains, the ORF50 locus, encoding the lytic regulator RTA, was also analysed.

### ORF50

**Fig 1:**
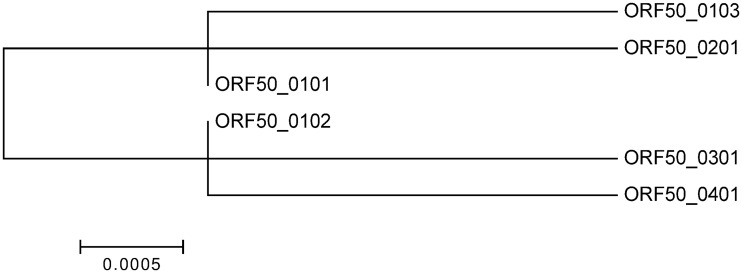
Phylogenetic analysis of AlHV-1 ORF50 gene sequences. Nucleotide sequences of the six variants ORF50*0101 to ORF50*0401 were aligned using MAFFT and phylogenetic analysis was done in MEGA version 6.0 using the Maximum Likelihood method, based on the Kimura 2-parameter model [38]. The tree with the highest log likelihood is shown. There were a total of 503 positions in the final dataset. The tree is drawn to scale, with branch lengths measured in the number of substitutions per site.

Amplification of ORF50 was attempted from all positive wildebeest samples (placenta and FTA card preserved buffy coat) and from FTA card preserved cattle samples (Table 1). For all FTA card preserved buffy coat samples, amplification was unsuccessful. In each case the amplification was repeated to confirm the result. ORF50 was successfully amplified from 38 of the 50 AlHV-1-positive placenta samples (S2 Data). Sequencing of these PCR products identified six distinct ORF50 alleles (Table 2) that shared more than 99% sequence identity. The majority of positive samples had a sequence identical to ORF50*0101 [31] (the allele carried by the C500 AlHV-1 strain). The other five alleles had ORF50 sequences that differed at only one or two base positions from ORF50*0101 (Fig 1 and S1 Fig). Two of the novel alleles (termed ORF50*0102 and ORF50*0103) encoded ORF50 proteins with identical sequence to ORF50*0101, whereas the remaining three novel alleles (0201, 0301 and 0401) encoded ORF50 proteins that differed by one amino acid from ORF50*0101 and by two amino acids from each other (S1 Fig).

**Table 2:** Summary of AlHV-1 genotyping results. (Sample type BC = Buffy Coat; FTA = stored on FTA cards).

Species	Wildebeest				Cattle
Provenance	Ngorongoro Crater			Tarangire	Tarangire
Age		Dam	Calf *	Calf **	Adult
Sample type	Placenta	BC FTA	BC FTA	BC FTA	BC
**Sample numbers**	94	3	5	5	6
**A9.5 alleles**					
A9.5*0101	13 (56%)	0	1	1	3
A9.5*0201^$^	0	0	0	0	0
A9.5*0202	0	0	0	0	3
A9.5*0203	5 (22%)	0	1	0	0
A9.5*0301	5 (22%)	0	1	0	0
**ORF50 alleles**					
ORF50*0101	29 (76%)	0	0	0	NA
ORF50*0102	5 (13%)	0	0	0	NA
ORF50*0103	1 (2.6%)	0	0	0	NA
ORF50*0201	1 (2.6%)	0	0	0	NA
ORF50*0301	1 (2.6%)	0	0	0	NA
ORF50*0401	1 (2.6%)	0	0	0	NA
**A9.5-ORF50 haplotypes**					
A9.5*0101 & ORF50*0101	7 (41%)	0	0	0	NA
A9.5*0101 & ORF50*0102	3 (17.6%)	0	0	0	NA
A9.5*0101 & ORF50*0301	1 (5.9%)	0	0	0	NA
A9.5*0203 & ORF50*0101	3 (17.6%)	0	0	0	NA
A9.5*0301 & ORF50*0101	3 (17.6%)	0	0	0	NA

* Calves < 30 days old

** Calves < 100 days old

^$^ As previously described [24]

### A9.5

**Fig 2:**
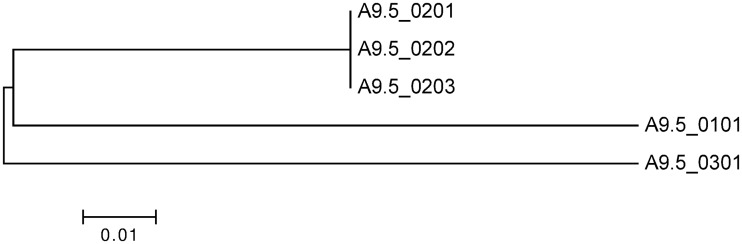
Phylogenetic analysis of A9.5 predicted cDNA sequences. Nucleotide sequences of the six variants A9.5*0101 to Ov9.5*0301 were aligned using MAFFT and phylogenetic analysis was done in MEGA version 6.0 using the Maximum Likelihood method, based on the Kimura 2-parameter model [38]. The tree with the highest log likelihood is shown. The tree is drawn to scale, with branch lengths measured in the number of substitutions per site. All positions containing gaps and missing data were eliminated. There were a total of 453 positions in the final dataset.

A9.5 could be amplified from 26 of 57 AlHV-1 positive wildebeest samples (three FTA card preserved buffy coat samples and 23 placenta) and from six of 59 AlHV-1 positive vaccine trial cattle samples (Tables 1, 2 and S1 Data). Four alleles of A9.5 were found among these samples, sharing at least 87% nucleotide sequence identity (S2 Fig). The majority of samples carried the A9.5*0101 allele, whilst the remaining samples carried three alleles which have not been previously described. These novel alleles were named, in accordance with the previously published nomenclature, A9.5*0202, A9.5*0203 and A9.5*0301. The novel alleles A9.5*0202 and A9.5*0203 encoded identical proteins to the previously published A9.5*0201 allele [24], differing only at two positions in the last intron. Novel allele A9.5*0301 encoded a protein sequence with 80% identity with the previously published alleles [24]. A9.5*0203 and *0301 were found only in wildebeest from the Ngorongoro Crater population, whilst A9.5 *0202 was only found in cattle living near Tarangire National Park (Table 2). The number of samples with these minority alleles was too low to allow statistical analysis. Phylogenetic analysis of the A9.5 genomic and putative spliced cDNA sequences yielded trees with the same topology (Fig 2).

**Fig 3:**
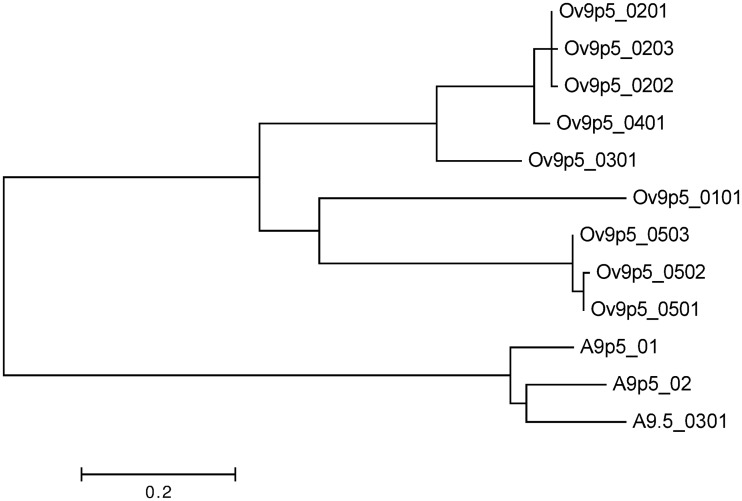
Phylogenetic analysis of A9.5 and Ov9.5 predicted protein sequences. Nine distinct Ov9.5 and three distinct A9.5 amino acid sequences were aligned using the Maximum Likelihood method based on the Whelan And Goldman model [41]. The tree with the highest log likelihood is shown. The tree is drawn to scale, with branch lengths measured in the number of substitutions per site. All positions containing gaps and missing data were eliminated. There were a total of 145 positions in the final dataset.

**Fig 4:**
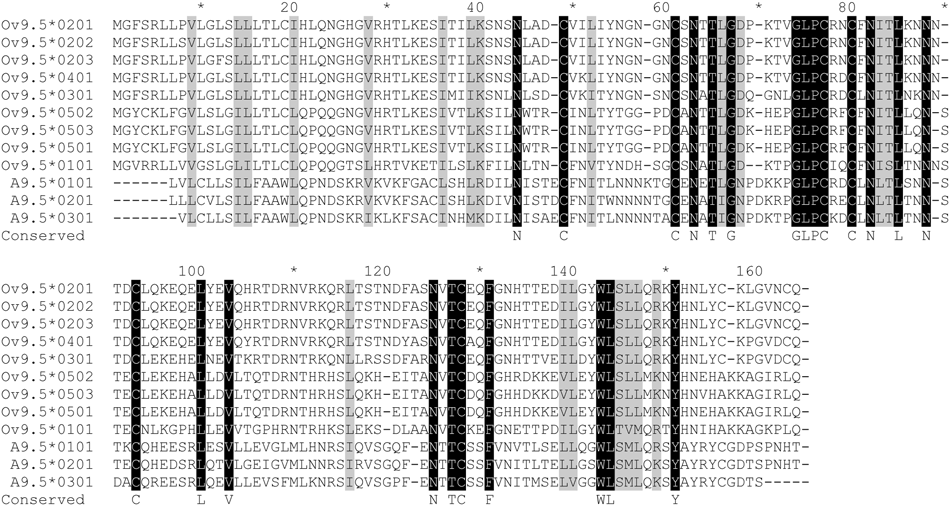
Alignment of A9.5 and Ov9.5 predicted protein sequences. The alignment was done by the Muscle algorithm [35] and conserved residues are highlighted as follows: residues identical in all sequences are shown in light face on black background; residues of conserved type (hydrophobic/charged/polar) are shown in black face with grey high-lighting. Residues identical in all sequences are also shown below the alignment on the line labeled ‘conserved’.

To study possible functional consequences of sequence diversity, the A9.5 protein sequences were compared with previously established Ov9.5 sequences [25]. Phylogenetic analysis indicated that the A9.5 alleles were less diverse than the Ov9.5 alleles (compare branch lengths in Fig 3). Within the protein sequence alignment, only 24 of 175 aligned residues were identical in all sequences, with a further 20 residue positions showing conservation of amino acid properties (size, charge, hydrophobicity). The fully conserved residues included six cysteines and five potential N-linked glycosylation sites (Fig 4).

The protein sequences aligned in Fig 4 were used to perform structure-based homology searching using the hhpred server [http://toolkit.teubingen.mpg.de/hhpred]. The top hit was interleukin-4 (*p* = 3.2x10^-5^), with similarity extending over the majority of the mature polypeptide (residues 26 to 151 of the alignment). This is in accord with previous suggestions that the A9.5 and Ov9.5 proteins share structural similarities with four-helix-bundle cytokines, in particular IL-4 [24] [25].

### Virus haplotypes

For both ORF50 and A9.5 the most frequent allele detected among animals characterized was the *0101 allele, the variant carried by the C500 strain of AlHV-1. In the 17 samples that could be genotyped at both loci, the most common haplotype was also that carried by the C500 virus (Table 2). All of the other combinations of ORF50 and A9.5 alleles that were detected contained either the ORF50*0101 or the A9.5*0101 allele in combination with a different allele at the second locus (Table 2).

## Discussion

In this study we have shown for the first time that AlHV-1 viral DNA can be detected in about 50% of placentae shed by parturient wildebeest. However, the observation that only about 1% of the same samples had virus detectable by real-time PCR suggests that the viral load in the wildebeest placentae tested was very low. There are two likely sources of the detected virus: i) latently infected cells from the dam circulation, which cannot be transmitted in these conditions. This would indicate that the placenta do not play a role in transmission of MCF; and / or ii) cell-free infectious virus from the calf’s circulation, which would support the view that these tissues may play a role in the transmission of virus to cattle, as considered by Massai pastoralists [21]. Further work to determine which of these two viral states predominate is required before conclusions regarding the role that placenta play in transmission can be drawn. The finding that many wildebeest placenta were infected, however, does support observations that wildebeest calves can be infected *in utero* [3],[15],[22].

Among the wildebeest tested, all five of the 32-day old calves from the Ngorongoro Crater population were positive for AlHV-1 by nested PCR, whilst only one of five 100-day old calves from the Tarangire population and none of the Ngorongoro Crater dams tested positive. However, ELISA testing showed evidence of AlHV-1 specific antibodies in all of the wildebeest tested except one calf in the youngest group. This suggests MCF virus-specific antibodies develop as the calves control the primary infection. These observations together support the view that infection of most wildebeest calves occurs *in utero* or within the first month of life. Furthermore, these observations are in accord with previous studies indicating that excretion of AlHV-1 by wildebeest calves peaks between one and two months of age and thereafter falls. By six months of age, despite being persistently infected, wildebeest have a very low level of circulating virus-infected cells (undetectable even by the sensitive nested PCR assay) and, consistent with latent infection, may no longer be capable of transmitting the virus to cattle [15],[39],[40].

Despite the identification of five new alleles, the ORF50 gene was highly conserved within the samples analysed, with the protein sequences encoded being identical or differing by up to two amino acids. In contrast, three new A9.5 alleles were characterised, and these encoded proteins that differed by approximately 20% of residues. It is notable that the A9.5 alleles differed from each other by less at the nucleotide level (9–13%) than at the amino acid level (19–20%). This is due to the presence of approximately two-thirds of the allelic differences in the exons and the occurrence of non-synonymous substitutions in more than half of the affected codons. This is suggestive of positive selection for variation in A9.5 but no clear evidence is available to support this [24].

Therefore, variation at the A9.5 locus might not be representative of variation across the whole virus genome. Indeed, while ORF50 is conserved, the A9.5 gene, as has been suggested for Ov9.5 [25], appears to be subjected to selective pressures that result in the polymorphism seen. In OvHV-2, nine different alleles of Ov9.5 have been classified [25], compared to the five A9.5 alleles (Fig 2). The Ov9.5 alleles are more divergent from one another, with 50% amino acid identity between the most divergent alleles, compared to the A9.5 gene in which the most divergent alleles have 80% predicted amino acid identity (Fig 3). The strong conservation of cysteine residues and potential glycosylation sites across all of the A9.5 and Ov9.5 alleles (Fig 4) suggests that this highly variable protein has conserved structure and function. Recent analysis of the A9.5 and Ov9.5 predicted protein sequences indicated similarity with the four-helix bundle cytokines, IL4 and IL21 [25]. Structural homology searching performed with the new A9.5 alleles identified in this study adds power to this analysis, identifying IL4 as the major homologue with a *p*-value enhanced from the earlier study. It is currently unclear whether the similarity with IL4 indicates only a shared structural fold or additional functional similarity.

Among 33 samples for which A9.5 sequences could be derived, only the most frequent allele, A9.5*0101, was found in wildebeest samples from both locations and in samples from cattle. A9.5*0202 was found only in cattle samples collected near Tarangire National Park, while A9.5*0203 and A9.5*0301 were detected only in wildebeest samples from the Ngorongoro Crater population. These geographical and species related differences may simply be a consequence of the low number of samples analysed from each site but may also result from the known isolation of the two wildebeest populations [28]. Unfortunately, ORF50 could only be amplified from wildebeest placental samples, so it is not possible to make any inferences from this data. It will be of interest to gather larger numbers of samples from across the range of wildebeest-associated MCF to determine the overall extent of AlHV-1 diversity and the epidemiology of MCF viruses in reservoir and susceptible species.

Herpesviruses, being DNA viruses, are typically stable with low rates of nucleotide substitution, as evidenced by the small numbers of base substitutions found among the ORF50 alleles sequenced. Thus, the alleles we have identified are likely to have been conserved in the population for a long period of time. This finding is supported by the fact that the previously characterized A9.5 alleles present in the C500 virus strain and in a diagnostic sample from wildebeest-associated MCF in the UK [27] encode A9.5 polypeptides that were identical to MCF virus fragments amplified from the cattle and wildebeest sampled in the two locations in Tanzania. Indeed, the most frequent haplotype identified among the Tanzanian samples was identical to the C500 virus at both the A9.5 and ORF50 loci, whilst all other haplotype combinations identified included *either* ORF50*0101 or A9.5*0101. This suggests that, like other herpesviruses which have relatively slow evolutionary molecular clocks [42], the C500 strain of AlHV-1 has remained closely related to AlHV-1 strains circulating in Tanzania, despite it having been propagated in African and European laboratories for approximately 50 years. This supports the view that C500 AlHV-1 remains a suitable strain for the development of a vaccine for wildebeest-associated MCF. Furthermore, similarity between the vaccine virus and Tanzanian strains of AlHV-1 reduces the risk of a novel, more pathogenic, virus mutant (derived by recombination between the vaccine virus and local strains of AlHV-1) escaping into the wild. This makes the AlHV-1 C500 strain safe for vaccination in MCF-endemic areas.

In summary, we have shown that about 50% of wildebeest placentae tested contained detectable AlHV-1 virus (measured as virus DNA), supporting evidence that wildebeest can be infected *in utero*, and suggesting that placenta could play a role in MCF transmission. The higher AlHV-1 DNA detection rate in the younger cohort of wildebeest calves is consistent with previous observations that infection of wildebeest generally occurs within the first month of life and that viraemia peaks between one and two months. The discovery of new A9.5 alleles supports previous evidence that the gene is highly polymorphic and appears to encode a secretory protein with IL-4 as the major homologue. Differences in the alleles detected in Tarangire and Ngorongoro Crater suggest that geographical and evolutionary separation of wildebeest populations [28] may have influenced the AlHV-1 alleles found. In contrast, the observation that the most frequently detected Tanzanian AlHV-1 haplotype was identical to the C500 virus suggests that AlHV-1 C500 is an appropriate strain for vaccine development and supports the view that AlHV-1 virus genotypes are stable over time. More extensive collection of samples from across the wildebeest range will enable the diversity of AlHV-1 strains to be further elucidated.

## Supporting Information

S1 Data The full set of A9.5 gene sequence data. The gene was successfully amplified from 26 AlHV-1 positive wildebeest samples and six AlHV-1 positive vaccine trial cattle samples
(TXT)

S2 Data The full set of ORF50 gene sequence data. The gene was successfully amplified from 38 AlHV-1-positive placenta samples
(TXT)

S1 Fig Alignment of the sequences of ORF50 PCR products determined in this work. The ORF50*0101 allele has sequence identical to that in the AlHV-1 genome sequence [31]. Nucleotide differences from the ORF50*0101 sequence are shown by grey highlights. The order of sequences in the alignment is optimised to reflect the major clades in Fig 1 defined by the C-T polymorphism at position 457. The three-letter translation of ORF50*0101 is shown below the alignment, with residues altered by base substitutions in alleles ORF50*0201, *0301 and *0401 indicated by underlining.
(TIFF)

S2 Fig Alignment of the sequences of A9.5 PCR products determined in this work. The positions of exons (uppercase) and introns (lowercase) were defined by comparison with the A9.5*01 sequence [24]. The flanking sequences in the A9.5*0101 allele that were used as primers are derived from the AlHV-1 genome sequence^31^ and are underlined. In the other alleles, only nucleotides that differ from the A9.5*0101 sequence are shown, while identities are represented by dots (.). Gaps, inserted to maintain alignment, are shown as dashes (-) and are found only in the introns.
(TIFF)
