# Supplementary figures and images for: Alcelaphine Herpesvirus-1 (Malignant Catarrhal Fever Virus) in Wildebeest Placenta: Genetic Variation of ORF50 and A9.5 Alleles
Source: PLoS One. 2015 May 13;10(5):e0124121. doi: 10.1371/journal.pone.0124121 (PMC4430166; doi:10.1371/journal.pone.0124121)

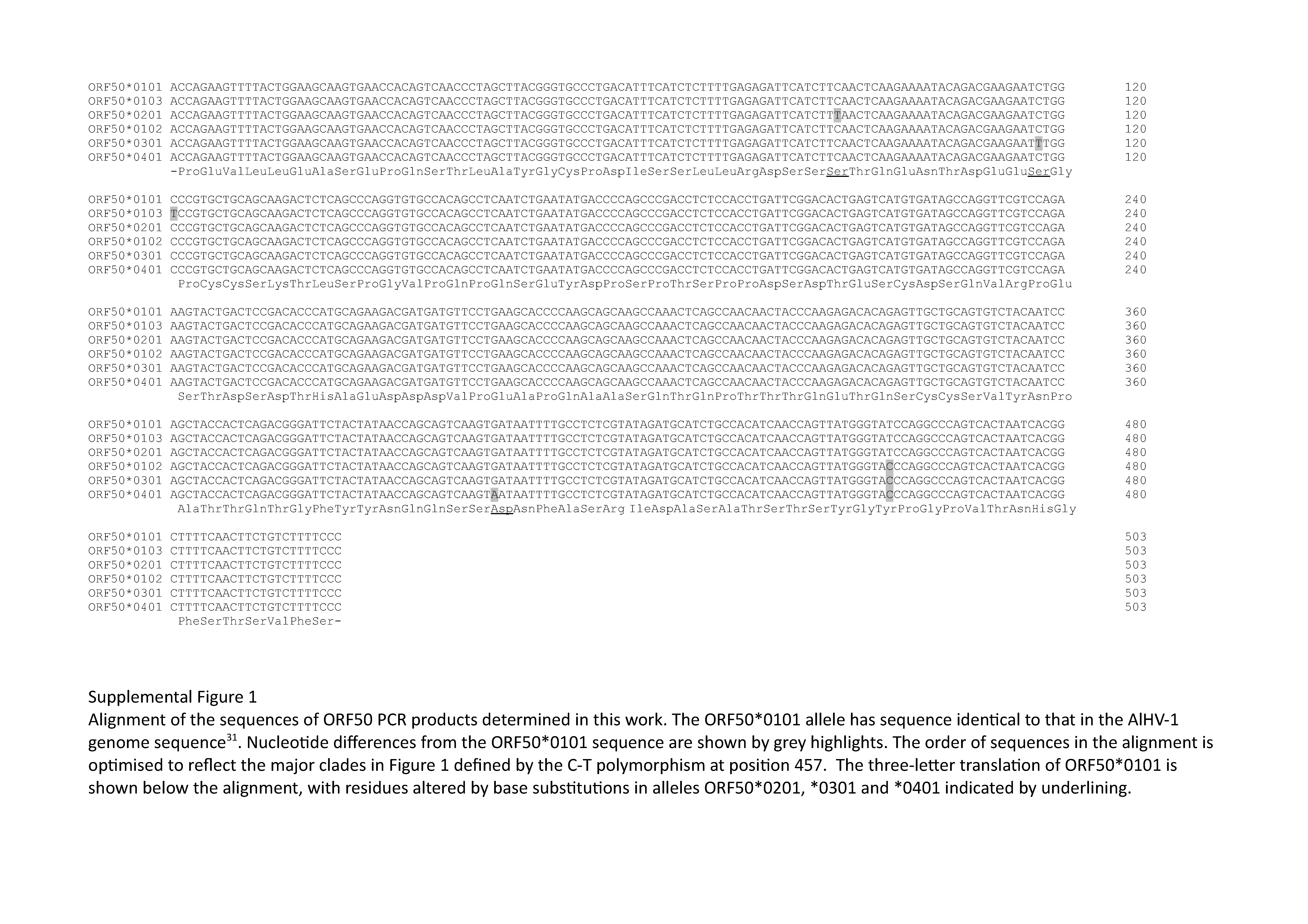

Supplement: S1 Fig — The ORF50*0101 allele has sequence identical to that in the AlHV-1 genome sequence [31]. Nucleotide differences from the ORF50*0101 sequence are shown by grey highlights. The order of sequences in the alignment is optimised to reflect the major clades in Fig 1 defined by the C-T polymorphism at position 457. The three-letter translation of ORF50*0101 is shown below the alignment, with residues altered by base substitutions in alleles ORF50*0201, *0301 and *0401 indicated by underlining. (TIFF) [file pone.0124121.s003.tiff]

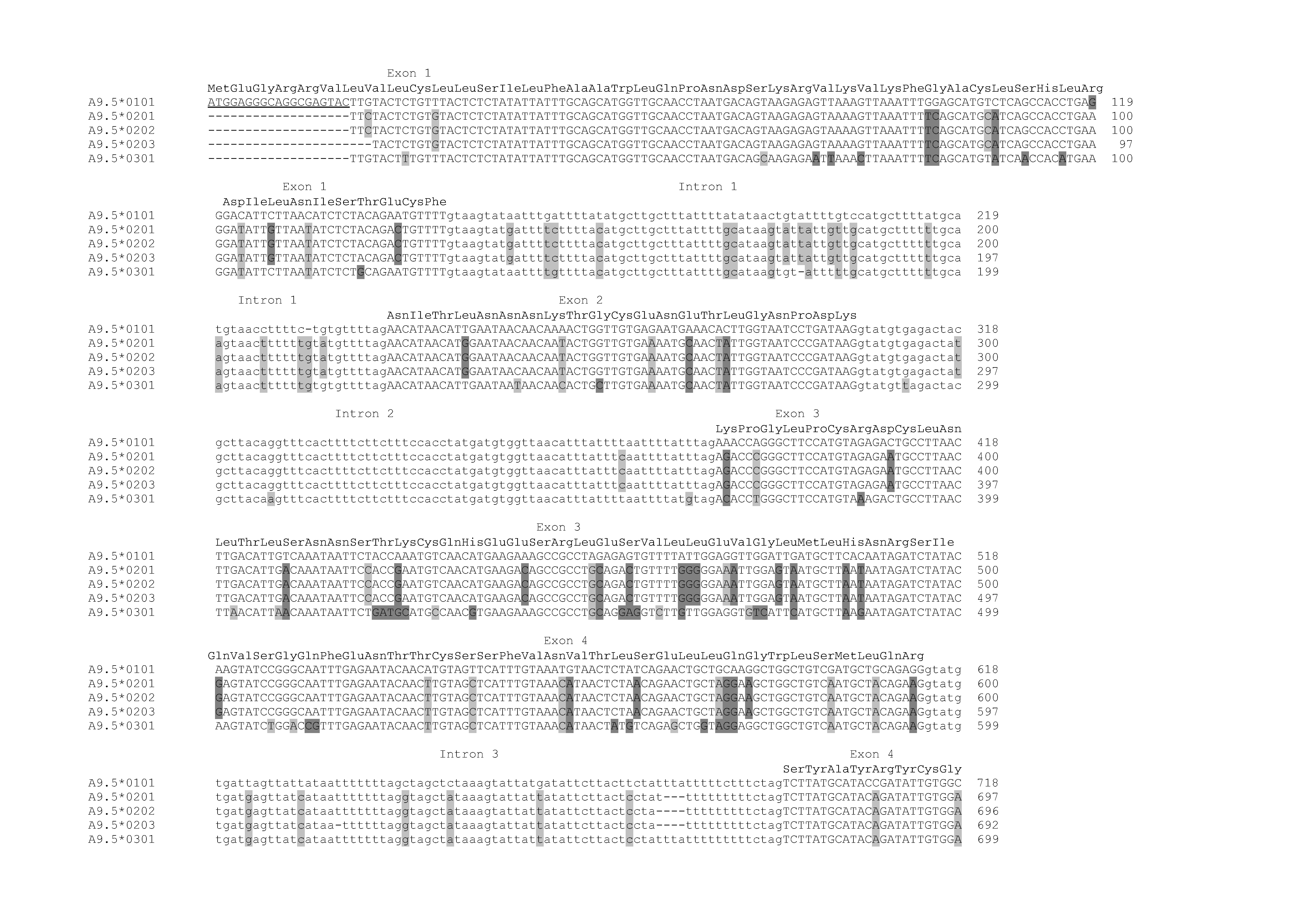

Supplement: S2 Fig — The positions of exons (uppercase) and introns (lowercase) were defined by comparison with the A9.5*01 sequence [24]. The flanking sequences in the A9.5*0101 allele that were used as primers are derived from the AlHV-1 genome sequence31 and are underlined. In the other alleles, only nucleotides that differ from the A9.5*0101 sequence are shown, while identities are represented by dots (.). Gaps, inserted to maintain alignment, are shown as dashes (-) and are found only in the introns. (TIFF) [file pone.0124121.s004.tiff]
